# Automatic Identification of Myeloperoxidase Natural Inhibitors in Plant Extracts

**DOI:** 10.3390/molecules27061825

**Published:** 2022-03-11

**Authors:** Fátima A. R. Mota, Sarah A. P. Pereira, André R. T. S. Araújo, Beatriz Gullón, Marieta L. C. Passos, Maria Lúcia M. F. S. Saraiva

**Affiliations:** 1 LAQV, REQUIMTE, Laboratory of Applied Chemistry, Department of Chemical Sciences, Faculty of Pharmacy, Porto University, Rua Jorge Viterbo Ferreira, No. 228, 4050-313 Porto, Portugal; fatimaamota@gmail.com (F.A.R.M.); sarah.pp@hotmail.com (S.A.P.P.); andrearaujo@ipg.pt (A.R.T.S.A.); 2 Unidade de Investigação para o Desenvolvimento do Interior, Instituto Politécnico da Guarda, Avenida Dr. Francisco de Sá Carneiro, No 50, 6300-559 Guarda, Portugal; 3 Department of Chemical Engineering, Faculty of Science, Universidade de Vigo (Campus Ourense), As Lagoas, 32004 Ourense, Spain; bgullon@uvigo.es

**Keywords:** myeloperoxidase, infection and inflammation, enzyme inhibition, plant extracts drugs, automation, sequential injection

## Abstract

The aim of this study is the development of an automated method for myeloperoxidase activity evaluation and its application in testing the inhibitory action of different plant extracts on the activity of the enzyme. This enzyme has its concentration increased in inflammatory and infectious processes, so it is a possible target to limit these processes. Therefore, an automatic sequential in-jection analysis (SIA) system was optimized and demonstrated that it is possible to obtain results with satisfactory accuracy and precision. With the developed method, plant extracts were studied, as promising candidates for MPO inhibition. In the group of selected plant extracts, IC50 values from 0.029 ± 0.002 mg/mL to 35.4 ± 3.5 mg/mL were obtained. *Arbutus unedo* L. proved to be the most inhibitory extract for MPO based on its phenolic compound content. The coupling of an automatic SIA method to MPO inhibition assays is a good alternative to other conventional methods, due to its simplicity and speed. This work also supports the pharmacological use of these species that inhibit MPO, and exhibit activity that may be related to the treatment of infection and inflammation.

## 1. Introduction

Early treatment of inflammatory and infectious points can greatly contribute to the prevention of progression to more severe clinical conditions. However, this is still challenging, due to all the problems inherent in the process [1,2,3,4,5,6].

Among the enzymes that intervene in these clinical processes, myeloperoxidase (MPO) is one of the most relevant enzymes in the context of inflammation and infection. MPO has a central role in the development of the infectious response, and in these sit-uations, MPO concentration levels are increased. This enzyme contains a heme group in myeloid cells (polymorphonuclear leukocytes (PMNs) and monocytes). PMNs are the first cells to reach the site, and, therefore, MPO is one of the first enzymes to appear, responding from the beginning of the infection in an acute and almost immediate way. Once neutrophils are recruited and stimulated, the increase in MPO concentration is also associated with the production of reactive oxygen species (ROS), reactive nitrogen species (RNS) and HOCl, which is the most powerful neutrophil oxidant. These compounds are very cytotoxic and react quickly with biological molecules, leading to protein degradation and inactivating enzymes. Thus, these species participate in inflammatory and infectious processes, and it is essential that their production is prevented [7,8].

The MPO is considered a possible target for drugs with small-molecule inhibitors to limit the production of the reactive species mentioned above [7]. The search for enzyme inhibitors through natural compounds is a strategic research and drug discovery jobs in research organizations around the world [9]. Plants applied in traditional medicine can provide numerous secondary metabolites with pharmacological potential as an anti-inflammatory and antioxidant, particularly phenolic compounds, flavonoids, and tannins [10].

In terms of enzyme inhibition, studies report that the natural compounds extracted from plant extracts had an inhibitory effect on the enzymes cathepsin G, human neutrophil elastase, cyclooxygenase, and xanthine oxidase [11,12,13,14,15]. However, considering the anti-inflammatory and antioxidant properties demonstrated by the extracts under study, it is desirable to explore the inhibitory effects on MPO activity [10].

Many non-steroidal anti-inflammatory drugs, anilines and phenols reversibly inhibit MPO. Anti-inflammatory drugs described as inhibitors of this enzyme have, in their structure, phenols or similar structures, which led us to further investigate these compounds’ ability to inhibit MPO [16,17]. These compounds could suppress ROS/RNS formation through the inhibition of certain enzymes, or metal-chelating compounds involved in the formation of free radicals. This situation is due to the fact that there are hydroxyl groups (-OH), in its structure, which are good H-donating antioxidants that interrupt the cycle of generating new radicals, which are formed with the increase in the MPO enzyme, due to inflammation [18]. Flavonoids are of particular interest due to their high antioxidant activity, and ability to act as hydrogen donors, reducing agents and suppressors of singlet oxygen or decaying peroxides [9].

Phenolic compounds are the most-studied natural compound present in plant extracts due to their beneficial properties for health, as has been demonstrated in many studies [19,20,21,22,23,24]. For example, extracts of Gentiana lutea proved to have a strong inhibitory effect on the enzyme, mainly its constituent gentiopicroside (IC_50_ = 0.8 µg/mL) [10]. In addition to this, other proven extracts demonstrated an inhibitory effect, such as extracts from Amazonian plants, where high levels of total polyphenols were related to antioxidant potential [9]. Studies indicate that the higher the content of polyphenols in the extracts, the greater the efficient inhibitory effect on the production of ROS [25]. Flavonoids are also widely described as the group of compounds that inhibit a series of enzymes, such as phosphodiesterase, Ca^2+^ ATPase, aldose reductase, lipoxygenase, cyclooxygenase, and human P450 [26]. In addition to all these characteristics, natural compounds have excellent advantages, as they are more innovative and economic, and have simple accessibility and reduced toxicity [27].

In this experimental work, we developed and optimized a methodology based on a sequential injection analysis (SIA) for the study of the inhibition of the enzyme myeloperoxidase with novel natural plant extracts. The selected plants were: *Foeniculum Vulgare* Mill., *Laurus Nobilis* L., *Citrus Sinensis*, *Camellia Japonica* L., *Rosmarinus officinalis* L., *Acacia dealbata* Link, *Aloysia citriodora* Palau, *Arbutus unedo* L. and *Genista tridentata* L. The extracts tested were mainly obtained from the leaves. They were selected because they are predominantly of Mediterranean geographic distribution, and after considering existing studies on each of them. Although some batch studies have already been conducted with other kinds of plants [9,10,25,28,29,30,31], the species evaluated herein have never been analyzed. Other studies are described for the extracts presented in this work [32,33,34,35,36,37], however, no automatic method was reported. Furthermore, there are no similar studies evaluating the inhibitory capacity of enzymes using natural plant extracts, and an automatic methodology such as SIA. The novelty and originality of this study are essentially based on the two criteria mentioned above: the specificity of the extracts and the methodology.

This is an automatic flow method, which allows for remarkable control of the reaction conditions, providing a useful tool for optimizing enzymatic reactions, enabling the same time elapse in all assays. Furthermore, these systems have numerous advantages when compared to discrete-batch mode methods. The SIA systems are highly versatile and robust, reduce operator intervention throughout the process due to computerized control, minimize reagent consumption and allow for minimal waste production [38].

## 2. Results and Discussion

**Figure 1:**
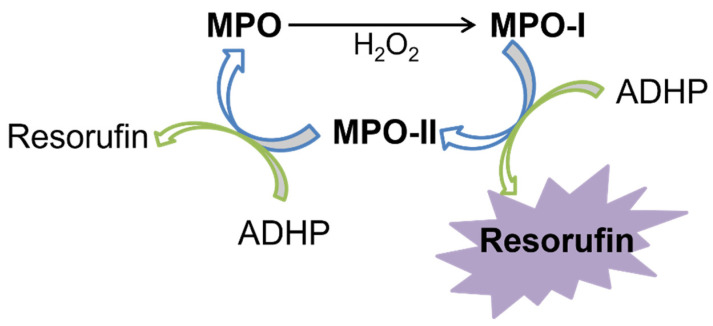
Enzyme reaction scheme of myeloperoxidase.

The development of the SIA system concept for the automatic establishment of MPO enzyme inhibition and the analysis of a set of plant extracts was proven by the reduction in enzyme activity using a fluorometric assay. This peroxidation reaction uses the enzyme MPO, to oxidize two electrons from the native enzyme (MPO) to MPO-I (compound I), through the action of H_2_O_2_. Then, there are two successive reduction reactions of an electron that returns to the native enzyme by MPO-II (compound II), using ADHP as substrate. The reaction between hydrogen peroxide and ADHP produces a highly fluorescent resorufin compound. The excitation and emission wavelengths defined for the resorufin measurement were 530 nm and 590 nm, respectively. The schematic reaction of the MPO is shown in Figure 1.

### 2.1. SIA System Optimization

To develop the method, firstly, physical, and chemical factors capable of affecting the reaction conditions were evaluated. The optimized parameters included pH, concentrations and volumes of used reagents, division and order of aspiration of the aliquots, propulsion time to the reactor and stopped-flow time. The studied ranges and the selected values for each optimized parameter are presented in Table S1 of the Supplementary Material.

The first parameter to be optimized was pH, and, according to the literature [39], the optimum value was not consensual, so we tested different values. The pH 7.4 showed about a 35% greater increase in FI when compared to the pH 6 solution, and the solution with pH 8 originated a saturated FI. Through this system, the zones containing reagents are sequentially aspirated to the holding coil, and, subsequently, there is a reversal of flow, and they are propelled to the detector. Interdispersion is essential for the partial overlapping of the zones to occur with the posterior reaction. Mixing the different zones is also favored by reducing reagent volumes

H_2_O_2_ is the reagent used by the enzyme as a co-substrate for oxidation reactions. However, studies reveal that high concentrations of hydrogen peroxide can lead to substrate inhibition and enzyme inactivation, affecting enzyme kinetics and endpoint fluorescence. The selected H_2_O_2_ concentration was 2.1 mM, since and higher concentrations lead to a decrease in analytical signals. The H_2_O_2_ optimization graph can be found in Figure S1 of the Supplementary Material, where, for a concentration of 50 µM of ADHP, there is an increase of almost 50% between the lowest FI (9.8 mM of H_2_O_2_) and the highest (2.1 mM of H_2_O_2_). Its volume was tested for 10 and 30 µL, and for volumes above 10 µL, the FI dropped to half (from 1.31 (10 µL) to 0.68 (30 µL).

Under the same experimental conditions, the influence of MPO concentration and volume was studied. Regarding the concentration, 0.40 U/mL (a unit of enzyme activity is established as one capable of converting 1 µmol of substrate or the formation of 1 µmol of product in one minute) [40] was selected, and was studied in the interval from 0.30 to 0.60 U/mL. We can conclude that there is a linear relationship between FI and enzyme concentration. For any of the enzyme concentrations, substrate concentrations were not limiting, and although 0.60 U/mL of enzyme provides a higher FI, it is possible to compensate for this by using more substrate and fewer enzymes by obtaining an analytical signal with equally good linearity. This can be seen in Figure S2 of the Supplementary Material, where the MPO and ADHP concentration optimization graph is found. Regarding volume, between 30 and 50 µL, the analytical signals were very similar (0.47 and 0.50, respectively), forming a plateau in the curve. This means that, from 30 µL, there is not enough reagent for the reaction to occur. Thus, the volume of 30 µL was chosen for the following tests, as, in addition to being identical to the 50 µL, it implies lower enzyme consumption and still presents good linearity.

Regarding the ADHP concentration, which works as the reaction substrate, it was considered that the higher its concentration, the greater the FI obtained, since this FI is proportional to the amount of resorufin that is produced. The highest concentration of ADHP studied was selected (40 µM), considering the compromise between the concentration of enzyme/ADHP and the analytical signal, and as this is not a limiting concentration for the enzyme. In Figure S2 of the Supplementary Material, this optimization is represented. The volume of ADHP was studied for the range of 10 to 40 µL, and the volume of 40 µL was selected. This is because, from 20 (0.11) to 40 µL (0.42), there an increase of more than 50% in FI, and the chosen volume is in a linear interval.

The division and order of aspiration of the aliquots were also optimized, and the best results in terms of FI were obtained when the H_2_O_2_ solution was aspirated last, suffering less dispersion, and in direct contact with the MPO. If we compare this selected aspiration order with an FI of 0.52, with the order H_2_O_2_–ADHP–buffer–enzyme with an FI of 0.27, where the H_2_O_2_ is aspirated at the beginning, the analytical signal decreases by 52%. This is because the dispersion suffered by the buffer along the path, both at the time of aspiration and propulsion, is greater, so the signal is lower. Thus, the selected order was ADHP–buffer–enzyme–H_2_O_2_.

Different propulsion times to the reactor were tested: 15, 20 and 30 s. The propulsion time for the reactor allows us to improve and minimize the maximum amount of time spent in each analytical cycle. It is also through the propulsion time to the reactor that you can stop the reaction in the best zone of the reaction tube. Considering the trade-off between time spent on each test and the analytical signal, we can conclude that 20 s is clearly the best time to propel the reaction zone to the reactor.

Finally, the stopped-flow period was optimized aiming the contact time of the substrate with the enzyme, with a consequent increase in sensitivity minimizing the dispersion. Three different times were tested: 1, 3 and 5 min. When we increased the time to 5 min, there was a large increase in FI (about 45%), along with an increase in the slope of the curve. As a compromise between analytical signal and sampling rate, no higher times were tested and in subsequent tests assays, a 5-min stopped-flow period was implemented.

A calibration curve with the optimized final conditions of the enzymatic reaction was obtained, with different MPO concentrations (*n* = 6). This curve presents a linearity range from 0.040 to 0.80 U/mL MPO. The calibration curve was FI = (1.095 ± 0.020) C + (0.014 ± 0.009) with an R2 = 0.999, where FI corresponds to fluorescence intensity and C corresponds to MPO concentration in U/mL, respectively, whose confidence limit is 95% for intercept and slope. The detection and quantification limits were calculations of the values obtained 0.013 U/mL and 0.043 U/mL, respectively.

To assess the repeatability of the developed methodology, a relative standard deviation (RSD) of 2.3% was obtained by injecting 15 (*n* = 15) consecutive assays using the enzyme concentration of 0.40 U/mL.

### 2.2. Evaluation of Plant Extracts Compounds

Once all the parameters of the developed SIA system were optimized, we applied the system to different plant extracts and obtained the inhibition profiles of all tested compounds. The half the maximum concentration of inhibition (IC_50_), and the respective values are shown in Table 1.

**Figure 2:**
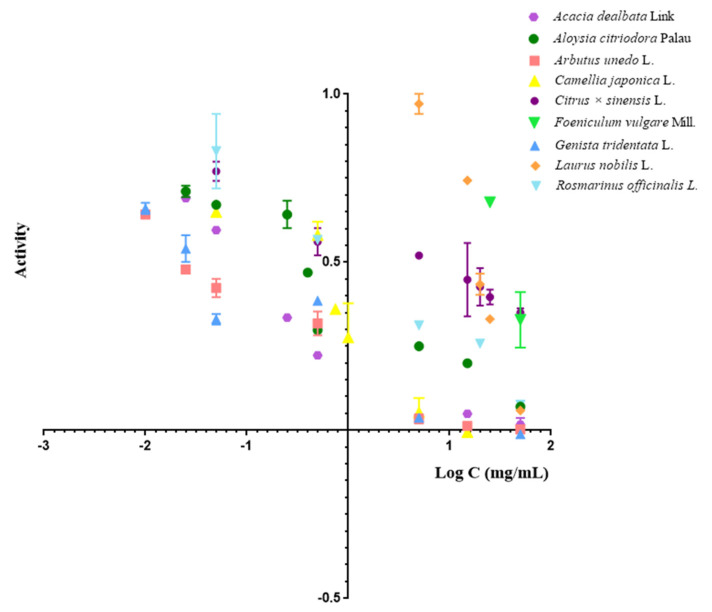
Inhibition profiles of plant extracts in MPO assays in SIA.

In Figure 2, the inhibition profiles of all extracts are graphically represented, and the individual inhibition profile of each tested extract is schematically presented in Figure S3 of the Supplementary Material. All compounds show a dose-dependent inhibition of MPO, and standard deviation (SD) values are lower than 3.5%.

The IC_50_ values described represent inhibitor concentrations that induce a 50% inhibition of MPO activity and were obtained from the analysis of inhibition curves in sigmoid form. Therefore, the lower the IC_50_ value, the lower the inhibitor concentration needed to inhibit 50% of the enzyme; therefore, this is more toxic for MPO.

Increased MPO concentration during inflammatory processes is related with the increased formation of reactive oxygen species and reactive nitrogen species during oxidative stress. The produced species are related to the oxidative explosion of neutrophils associated with tissue damage, which sustains the failures and anomalies that occur during inflammation. The phenols present in plant extracts have hydroxyl groups (-OH) in their structure, which are good hydrogen donors. These groups (-OH) can bind to the active site of the enzyme (preventing the binding with the substrate) and preventing the formation of the product, thus providing a competitive and reversible inhibition. When this happens, the ROS and RNS generation cycle will be interrupted through inhibition of the MPO enzyme. The antioxidant and anti-inflammatory potential of phenols is related to the presence of OH structures in their composition [16,17,18,41,42,43].

By critically analyzing the results in Table 1, it is possible to compare the different extracts. Regarding the extracts derived from *Foeniculum vulgare* Mill. and *Arbutus unedo* L., we realize that they are the ones with the highest and lowest IC_50_, and are accordingly the least and most toxic for MPO, respectively. Although no clear justification was previously studied, we can start from some studies that were previously carried out with these plants and draw some conclusions. If we check the phenolic composition of the species *Foeniculum vulgare* Mill., we can see that it is essentially constituted by phenolic acids, mainly chlorogenic, caffeic and rosmarinic acid, and by some flavonoids, mainly quercetin, rutin and apigenin [44,45,46]. However, the flavonoids are present in lower concentrations, and there are few subclasses of them. On the other hand, if we evaluate the species *Arbutus unedo* L., mostly flavonoids such as quercetin, anthocyanins (cyanidin and delphinidin glycosides), among others, are identified. In addition to these, hydroxybenzoic acids such as gallic acid were the main phenolic compounds reported and, in minor concentrations, chlorogenic acid was also found [47,48,49]. Flavonoids are able to modulate enzyme activity, and, for this reason, they are the main class of phenolic compounds that exhibit the strongest antioxidant activity. Therefore, it is justified that the species *Arbutus unedo* L. is more toxic to the enzyme and has the lowest IC_50_ [50,51,52]. Previous investigations have indicated that the bioactivities and binding affinities for proteins can be influenced by the patterns of the hydroxyl and methoxyl groups on the rings of flavonoids [26]. In addition to this justification, quercetin also demonstrated activity in the activation of the complement system, thus decreasing the call of inflammatory cells to the endothelium and, consequently, reducing the inflammatory response [50]. On the other hand, a plausible justification is also the presence of anthocyanin subclasses such as cyanidins (delphinidin-3-*O*-glucoside), which are of particular importance due to their peculiar nutraceutical properties. The antioxidant potential of the delphinidin glycosides showed stronger activity against the scavenging of superoxide anion and peroxynitrite among several anthocyanins. It has also been described to exert effects against oxidative stress and noted for its anti-inflammatory activity. As MPO present in inflammation, its inhibition by this compound is superior when compared to other extracts [46,53].

Considering the species *Laurus nobilis* L., we can prove that it has a lower IC_50_ than the species *Foeniculum vulgare* Mill., although a higher one than the others, and is also considered less toxic. Observing its composition [54,55], we can see that it is identical to the species *Foeniculum vulgare* Mill.: that is, mainly composed of phenolic acids such as caffeic acid, ferulic acid and chlorogenic acid, and has lower concentrations of flavonoids such as quercetin, kaempferol and apigenin than other species (but higher than the species *Foeniculum vulgare* Mill.). It should be noted that, despite not appearing in large concentrations, this species has a greater amount of gallic acid, and an important responsibility as an antioxidant [10,56]. Furthermore, quercetin and apigenin (flavonol and flavone, respectively), which are present in this species, increase the inhibitory activities within the flavonoid class. This is due to their greater binding affinity, and increased opportunities to enter and affect the enzyme’s catalytic site [26]. Thus, it is possible to justify the differences in the IC_50_ of the two species, and the high IC_50_ value with a lower toxicity [54,55].

In relation to the smallest IC_50_ observed in Table 1, that is, in relation to the most toxic extracts for MPO, we can compare the species *Arbutus unedo* L., which was previously analyzed, and *Genista tridentata* L. Previously, in the analysis of the species *Arbutus unedo* L., we noticed that the greater presence of flavonoids in plants in relation to other types of phenols was one of the justifications for its greater toxicity. Therefore, and considering that the two species mentioned above are the most toxic in terms of IC_50_, we can also detail the composition of the species *Genista tridentata* L. and understand why both have a low IC_50_. *Genista tridentata* L. is rich in taxifolin, ginestein, and ginstein derivatives, biochanin A-glucoside, biochanin A.: that is, this species has a high content of flavonoids, mainly isoflavones. The presence of taxifolin, ginestein and ginestatin is related to the antimicrobial, anthelmitic and antioxidant activities of the species, which makes it more relevant from a bioactive perspective. Taxifolin is a subclass of flavonoids that are abundant in several types of plants, and is mainly of interest for food supplements that are rich in antioxidants. On the other hand, ginestein is an isoflavone with anti-inflammatory and antioxidant properties, and this compound may be responsible for its use in traditional medicine. Quercetin has also been reported in the composition of this species, as well as in *Arbutus unedo* L. Although there is still no clear relationship between affinities and inhibition through flavonoids, there was an increase in inhibitory activities with increased affinities within the class of flavones and flavonols (quercetin). This can be justified since the higher the binding affinity, the greater the opportunity to enter and affect the enzyme’s catalytic site, while the inhibition was due to the direct interaction between the flavonoids and the active site [26]. In this way, it is possible to justify the low IC_50_ of this species [57,58,59,60].

As can also be seen in Table 1, some compounds have identical IC_50_s, such as *Rosmarinus officinalis* L. and *Citrus × sinensis*. It is possible to verify that, according to the IC_50_ values, the species *Rosmarinus officinalis* L. is more toxic to the enzyme. However, it is important to understand the small differences are that occur when the IC_50_s are so close. For this, it is important to compare the two species. In terms of flavonoid composition, both have Hesperidin as the major component [61,62]. In addition, luteolin, Apigenin, Genkwanin were found for the species *Rosmarinus officinalis* L. and diosmetin, naringin, tangeretin, quercetin, nobiletin and narirutin for *Citrus × sinensis*. Phenolic acids were found in two species, Gallic acid, Caffeic acid, Ferulic acid, Coumaric acid, caffeic acid, found in in higher concentrations in *Citrus × sinensis* and, naturally, rosmarinic acid (only present in *Rosmarinus officinalis* L. species), found in higher concentrations in the other species mentioned [61,62,63]. Typical phenolics with antioxidant activity are primarily flavonoids (as we have mentioned) and phenolic acids. Taking this into account, we can draw some conclusions regarding its toxicity. Although hesperidin is the major flavonoid in both species, it is found in higher concentrations in the species *Rosmarinus officinalis* L. than in *Citrus × sinensis*, which has more types of flavonoids; therefore, the concentrations are more dispersed. This may be a possible justification for the small difference in IC_50_ [61,62,63]. Furthermore, an important difference between these two compounds is the presence of rosmarinic acid in only one of the species. This phenolic acid is one of the most potent in terms of antioxidant activity, even when compared to quercetin (flavonoid). Within the compounds related to rosmarinic acid, the existence of the group ortho dihydroxy proved to be a relevant and critical structure, linked to the intrinsic antioxidant activity. This structure is also present in some rosmarinic acid derivatives, such as ferulic acid (also present in *Citrus × sinensis*), which demonstrates a comparable oxidizing activity. However, this antioxidant capacity in ferulic acid is modified by O-methylation, which makes it less active [61,62,63,64]. Thus, it is possible to understand why the species *Rosmarinus officinalis* L. has a lower IC_50_ than *Citrus × sinensis*.

As a critical and global analysis, the novelty of this study was established through the development of an automatic method to evaluate MPO inhibition with plant extracts. This method is highly innovative, and there is no report of its use for the referenced species, in addition to all the advantages mentioned above.

A positive control, an effective MPO inhibitor, called 4-Aminobenzohydrazide, was also tested. The IC_50_ value obtained was 24.8 ± 4.9 µM. If we compare this inhibitor with the others tested, we can see that the MPO inhibitor showed a higher IC_50_ for the enzyme than all other compounds, except *Foeniculum vulgare* Mill. In this way, the compounds of natural origin have a greater capacity to inhibit MPO and, therefore, more capacity to reduce wound infection.

The batch procedure was used to test some compounds and the respective IC_50_s obtained by the two methods are compared in Table 2.

As it is possible to verify the two methods, the increasing order of values and compounds is the same, that is:

1. *Arbutus unedo* L.
2. *Genista tridentata* L.
3. *Aloysia citriodora* Palau
4. *Camellia japónica* L.
5. *Rosmarinus officinalis* L.
6. *Laurus nobilis* L.
7. 4-Aminobenzohydrazide (positive control)
8. *Foeniculum vulgare* Mill.

The higher the IC_50_, that is, the higher the concentration needed to inhibit the enzyme, the more difficult it is to correlate the two methods, and there are more differences between the obtained values. This can be observed in Table 2, where, if we consider the *Foeniculum vulgare* Mill. Species, the difference between SIA and the batch is greater than for the *Camellia japónica* L. species, with a lower IC_50_ concentration, which shows fewer differences. This can also be explained by the fact that the concentrations and volumes of reagents used in the batch method are higher, which, in terms of the number of moles, is also higher. For example, in SIA, an ADHP concentration of 40 µM and a volume of 40 µL was used, which corresponds to 0.00016 moles, while, in batch, the concentration is 200 µM and the volume 125 µL, which equals 0.025 moles.

The compliance between the two methods was assessed using three statistical tests. The *t*-test was performed, as a double-sided coupled test; the tabulated *t* value was 2.37 and the calculated *t* was 0.0071. These results demonstrate that there are no statistically significant differences between the results obtained with the two methods, with a confidence level of 95%.

A linear relationship was also established, as described by the Equation (1):

**IC_50_ BATCH = (1.15 ± 0.45) IC_50_ SIA + (1.2 ± 7.5)** (1)

In the equation, IC_50_ BATCH and SIA represents the IC_50_ values that were obtained through the tested methods (BATCH and SIA), respectively, whose confidence limit is 95% for the intercept and slope. The calculated intercept and slope values do not differ substantially from 0 and 1, respectively, which proves the agreement between the SIA system and the BATCH method.

Regarding the Pearson correlation coefficient, it was calculated to be (~0.93) and shows that the two sets of results have a good correlation using the developed method and the batch method. The *p*-value was also calculated (*n* = 7), and the obtained value was 0.97, with a 95% confidence level (*p* = 0.05) and no statistical differences between the two methods.

## 3. Materials and Methods

### 3.1. Reagents

All the chemicals were of analytical grade and the solutions were prepared with ultrapure water from a Milli-Q water plus system (Millipore, Danvers, MA, USA)with specific conductivity of less than 0.1 µS cm^−1^.

Myeloperoxidase from human leukocytes (MPO), 10-Acetyl-3,7 dihydroxyphenoxazine also called Amplex^®^ Red (ADHP), hydrogen peroxide (H_2_O_2_), sodium phosphate dibasic (Na_2_HPO_4_) and sodium phosphate monobasic (NaH_2_PO_4_) were all supplied by Sigma-Aldrich^®^ (Sigma Aldrich St. Louis, MO, USA). Dimethylsulfoxide (DMSO) was used for the dissolution of ADHP and was supplied by Merck^®^ (Darmstadt, Germany).

The buffer solution (PBS) 0.20 mol/L (pH 7.4) was prepared, weighing 17.8 g of sodium phosphate dibasic (Na_2_HPO_4_) dissolved in about 500 mL of water. A 0.20 M sodium phosphate monobasic (NaH_2_PO_4_) solution was used to adjust the pH to 7.4 and the final volume was fixed to 1000 mL. of water. The PBS buffer solution was kept in the refrigerator.

To prepare an enzyme stock solution of 5 U/mL, 5 U of the commercial enzyme was dissolved in 1000 µL of PBS buffer. The final volume of the stock solution was divided into 20 µL per eppendorf, for a total of 50 eppendorf, which were then frozen. After defrosting, it had to be rejected at the end of the day and could not be frozen again.

The 300 μM ADHP solution was prepared dissolving a mass of 0.00030 g in a final volume of 4000 μL (360 μL of DMSO + 3640 μL PBS buffer). Subsequently, this volume was equally divided by 10 eppendorfs, which were frozen. A working solution of 40 μM was prepared by diluting 400 μL of the stock solution in a final volume of 3000 μL. To guarantee the same amount of DMSO in all ADHP solutions, the DMSO and the buffer were added proportionally. After preparation, it was deoxygenated with nitrogen gas, and then protected from light with aluminum foil.

A 2.1 mM H_2_O_2_ solution was prepared from the dilution of a 30% solution (9.8 mM). It should be noted that this solution was prepared daily.

For enzyme inhibition assays, the samples of plant extracts that were used were obtained from different plants through selective harvesting. Ten different plant extracts were tested, including: fennel (*Foeniculum vulgare* Mill.), laurel (*Laurus nobilis* L.), orange (*Citrus Sinensis*), japanese camellia (*Camellia Japonica* L.), rosemary (*Rosmarinus officinalis* L.), mimosa/acacia (*Acacia dealbata* Link), lemon verbena (*Aloysia citriodora* Palau), strawberry tree (*Arbutus unedo* L.) and Prickly broom (*Genista tridentata* L.).

4-Aminobenzohydrazide was used as positive control for the inhibition of enzyme activity.

### 3.2. Sample Treatment

The treatment of the samples was carried out to obtain the phenolic compounds present in the plant extracts. The plants were collected in the province of Ourense, Spain, and mainly the leaves were used. Each sample was extracted in an orbital shaker at 120 rpm, at 50 °C for 90 min. The solvent mixture used was 50% ethanol–water, and a plant–solvent ratio of 20 g/mL was selected. After a vacuum filtration was used to recover the extracts, they were stored in the dark at −20 °C until use.

At the moment of utilization, they were centrifuged at 10,000 rpm for 10 min at room temperature and the supernatant was collected. In addition to this, a filtration was performed using filters with a pore size of 0.22 µm, which are used to retain small particles. Thus, it was possible to remove all residues present in the sample. The resulting supernatant was analyzed in the SIA system, and directly or previously diluted in the mixture of H_2_O and ethanol (1:1).

### 3.3. Apparatus

**Figure 3:**
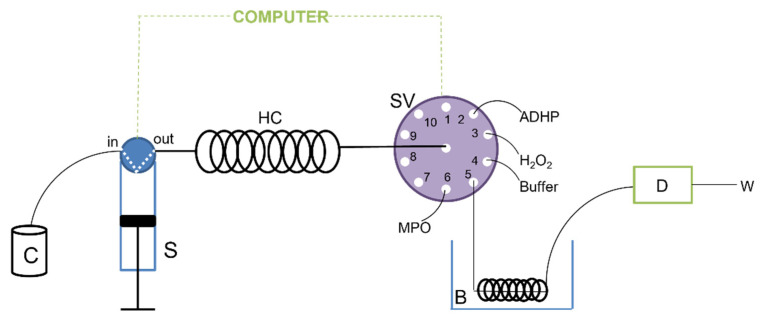
Schema representation of the developed SIA system. C: carrier; S: syringe; HC: holding coil; SV: selection valve; B: thermostatic bath (37 °C); D: detector; W: waste.

The SIA system developed to test the inhibition of the myeloperoxidase enzyme is illustrated in Figure 3. This system consisted of a Bu1S syringe module from Crison Instruments S.A. (Allela, Barcelona, Spain), a 10-port multi-position CheminertTM selection valve and a VarioMag^®^ (Port Orange, FL, USA) thermostatic bath. A solenoid valve, on the top of the syringe, allowed for the syringe to be switched to the manifold or carrier positions. The modules were controlled using a computer. Visual QuickBasic 4.5 software was used to control the instruments, and the communication between them was established through asynchronous RS-232C protocols, via embedded dynamic libraries. The computer allows for control of the flow direction, the flow rate, the valve position, the stopped flow period, the enzyme, substrate, and sample volumes, and synchronization between equipment and data acquisition. Manifold components were connected by tubes of polytetrafluoroethylene (PTFE) with 0.8 mm i.d., which was also used for the holding coil (2 m).

A fluorimeter from Jasco^®^ (Jascoinc., Easton, PA, USA), model FP-2020 Plus, was used to measure the fluorescence intensities (FI). The flow cell used had 16 μL. It was used with a gain of 16 and an attenuation of 100. The excitation and emission wavelengths were set at 530 and 590 nm, respectively.

To proceed with the extraction of the samples, an orbital shaker, Zhicheng HWY-111B (Zhichenginstrument, Shanghai, China)was used. For the centrifugation of plant extracts, a Beckman Coulter model Microfuge 22^®^ centrifuge was used. For the extracts, Filter-Bio^®^ (Nantong, China) filters with a pore size of 0.22 µm attached to a syringe from Braun Ominifix^®^ (Barcarena, Portugal) were used to filter natural compounds and retain small particles.

To perform the batch assays, a fluorimeter Jasco FP^®^ 6500 and a cell with a capacity of 700 µL from the Hellma^®^ (Hellma analytics, Kruibeke, Belgium) Analytics were used.

#### Sequential Injection Analysis Procedure

Through the SIA system, the enzymatic reaction of myeloperoxidase was carried out first and the effect of plant extracts on myeloperoxidase activity was then evaluated. Initially, all the system was filled with the PBS solution at pH 7.4, the carrier solution. The tubes from positions 2, 3, 4 and 6 were instead filled with the solutions of ADHP, H_2_O_2_, plant extracts and MPO, respectively.

An analytical cycle was defined for the enzymatic reaction of MPO, in the SIA system, as presented in Table S2 of the Supplementary Material. This cycle was performed by aspiration of 40 μL of ADHP, 10 μL of sample, 30 μL of enzyme and 10 μL of H_2_O_2_ (step 1, 2, 3 and 4). After this, the solution was propelled to the reaction tube (step 5), where the flow was stopped in the thermostatic bath for 5 min (step 6). Then, the mixture was propelled to the detector where the fluorescence of the formed product was measured (step 7).

The fluorescence intensity without inhibition is related to the maximum enzyme activity. Thus, after the application of plant extracts, the fluorescence intensity should decrease, due to the enzymatic inhibition caused by the extracts.

A blank measurement was carried out, with only ADHP and H_2_O_2_, without enzyme, under the same conditions, to exclude the intrinsic fluorescence effect of ADHP. The temperature of all assays was 37 °C and duplicates of each assay were performed.

The formula used to calculate inhibition by this method was:

(2) $$\%\;Inhibition=\frac{100\times \left(control\;reaction\left(without\;inhibitor\right)-reaction\;with\;inhibitor\right)}{control\;reaction}\;$$

### 3.4. Batch Procedure

The definition of the batch conditions was based on an MPO inhibitor screening assay kit from Abcam^®^ (Waltham, MA, USA) [39].

For each assay, we used a mixture of 25 μL of 0.40 U/mL of MPO, 2.5 μL to 3% of H_2_O_2_, 125 μL of 200 μM of ADHP and 97.5 μL of PBS buffer pH of 7.4, creating a final volume of 250 μL. The reaction was measured after 5 min at 37 °C. The excitation and emission wavelengths were 530 and 590 nm, respectively. Each assay was carried out in duplicate. The formula used to calculate inhibition by this method was the same as used for SIA methodology.

### 3.5. Statistical Analysis

Errors and standard deviations, just as all statistical tests were calculated throughout the work using the Excel tool for Windows (Microsoft). The IC_50_ was obtained by plotting the graphic regression using GraphPad Prism 7 software.

## 4. Conclusions

A sequential injection analysis system was studied and developed to assess the effect of plant extracts on the myeloperoxidase enzyme activity. The capacity of plants to produce new therapeutic products motivated research into the screening of new natural MPO inhibitors. Through this work, it was possible to confirm the inhibitory capacities on the myeloperoxidase enzyme, which can be an excellent tool to prevent the progression of inflammation or infection to more severe conditions, allowing it to act quickly and at an early stage in these processes. The ability to inhibit phenols in its composition is due to their antioxidant and anti-inflammatory capacity, which is inherent to the presence of hydroxyl groups in their structure that bind to the active site of the enzyme, competing with the substrate. The formation of the final product will be inhibited, as well as the formation of highly toxic species for the organism, which consequently aggravate the infectious and inflammatory processes. Flavonoids seems to exhibit antioxidant properties at a larger scale, and therefore, when present in high concentrations, have a greater capacity to inhibit the enzyme. This can be explained by its high affinity with proteins, and its ability to modulate enzymatic activity. Of all the extracts studied, *Arbutus unedo* L. was the most promising, in the sense of its being applied as a therapeutic agent in the inhibition of MPO, requiring low concentrations to inhibit 50% of the enzyme.

These automatic systems are very beneficial tools for carrying out enzymatic reactions related to MPO, such as an analysis of the inhibitory profile of plant extracts. The results obtained are reliable and trustworthy, proving the use of this system in future implementations. In addition, these automatic flow techniques, such as SIA systems, allow for precision of the techniques to be increased using the same volumes and times in all tests, improving the reproducibility of the method. Additionally, there is a reduction in the consumption of reagents, minimizing waste and reducing the need for operator intervention, due to the computerized control, which reduces operator failures.

Thus, the developed SIA method proved to be more advantageous in relation to the batch method, due to the reduced operator intervention, reduced sampling time, reduced volume of reagents and samples and ease in terms of clinical applicability.

As a future perspective, the main objective is the application of wound samples in the enzymatic reaction and the developed system. The main purpose is to be able to quantify the amount of MPO in each sample and understand the inflammation state of the wound from which it was taken, considering the reference values of the concentration of MPO in inflamed and non-inflamed wounds [2,65].

## Figures and Tables

**Table 1:** Different plant extracts tested for myeloperoxidase inhibition and their IC_50_.

Scientific Name of Plant Extract	Common Name of Plant Extract	IC_50_ (mg/mL) ± SD
*Acacia dealbata* Link	Mimosa; Acacia	0.086 ± 0.001
*Aloysia citriodora* Palau	Lemon verbena	0.282 ± 0.040
*Arbutus unedo* L.	Strawberry tree	0.029 ± 0.002
*Camellia Japonica* L.	Japanese camellia	0.303 ± 0.066
*Citrus × sinensis*	Orange	5.4 ± 3.1
*Foeniculum vulgare* Mill.	Fennel	35.4 ± 3.5
*Genista tridentata* L.	Prickly broom	0.032 ± 0.004
*Laurus nobilis* L.	Laurel	19.62 ± 0.28
*Rosmarinus officinalis* L.	Rosemary	1.02 ± 0.28

**Table 2:** Comparison of SIA and BATCH IC_50_ values.

Plant Extract	IC_50_ (mg/mL) Obtain in SIA ± SD	IC_50_ (mg/mL) Obtain in Batch ± SD
*Aloysia citriodora* Palau	0.282 ± 0.040	0.258 ± 0.029
*Arbutus unedo* L.	0.029 ± 0.002	0.004 ± 0.001
*Camellia Japonica* L.	0.303 ± 0.066	0.290 ± 0.083
*Foeniculum vulgare* Mill.	35.4 ± 3.5	50.6 ± 9.7
*Genista tridentata* L.	0.032 ± 0.004	0.044 ± 0.013
*Laurus nobilis* L.	19.62 ± 0.28	13.7 ± 1.4
*Rosmarinus officinalis* L.	1.02 ± 0.28	1.67 ± 0.29
4-Aminobenzohydrazide (positive control)	24.8 ± 4.9	17.4 ± 4.3

## Data Availability

Data is contained within the article or Supplementary material. The data presented in this study are available in this manuscript.

## Supplementary Materials

The following supporting information can be downloaded at: https://www.mdpi.com/article/10.3390/molecules27061825/s1, Table S1: Different conditions tested and optimization results of the SIA system developed.; Figure S1: Influence of H2O2 concentration on the analytical signal of the enzymatic reaction.; Figure S2: Influence of MPO and ADHP concentration on the analytical signal of the enzymatic reaction; Figure S3: Experimental inhibition data of the plant extracts tested in the MPO inhibition assays in SIA; Table S2: Analytical cycle developed and optimized in the SIA system.

## Sample Availability

All samples of plant extract were obtained by Beatriz Gullón.
