# Supplementary material for: Automatic Identification of Myeloperoxidase Natural Inhibitors in Plant Extracts
Source: Molecules. 2022 Mar 11;27(6):1825. doi: 10.3390/molecules27061825 (PMC8950977; doi:10.3390/molecules27061825)
Supplement: Supplementary file 1 [file molecules-27-01825-s001.zip › molecules-1608722-supplementary.pdf]

Supplementary material

Table S1. Different conditions tested and optimization results of the SIA system developed.

Parameter		Range	Selected value
pH		6; 7.4; 8	7.4
MPO	Concentration	0.30 U/mL; 0.40 U/mL; 0.60 U/mL	0.40 U/mL
	Volume	10 µL; 30 µL; 50 µL	30 µL
ADHP	Concentration	10 µM; 20 µM; 40 µM	40 µM
	Volume	10 µL; 20 µL; 30 µL; 40 µL	40 µL
H_2O_2	Concentration	9.8 mM- 0.26 mM	2.1 mM
	Volume	10 µL; 30 µL	10 µL
Division and order of aspiration of the aliquots		H_2O_2-ADHP-buffer-enzyme (◆) H_2O_2-buffer-enzyme-ADHP (■) ADHP-H_2O_2-buffer-enzyme (▲) ADHP-buffer-enzyme-H_2O_2 (×)	ADHP-buffer-enzyme-H_2O_2 (×)
Propulsion time to the reactor		15; 20; 30 seconds	20 seconds
Stopped flow period		1; 3; 5 minute	5 minutes

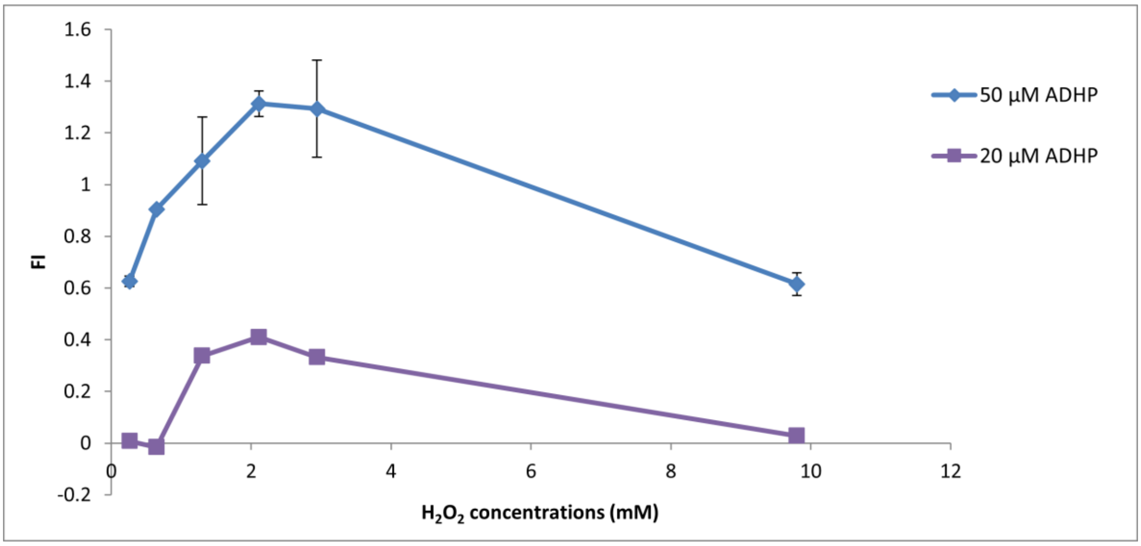

Figure S1. Influence of H_2O_2 concentration on the analytical signal of the enzymatic reaction.

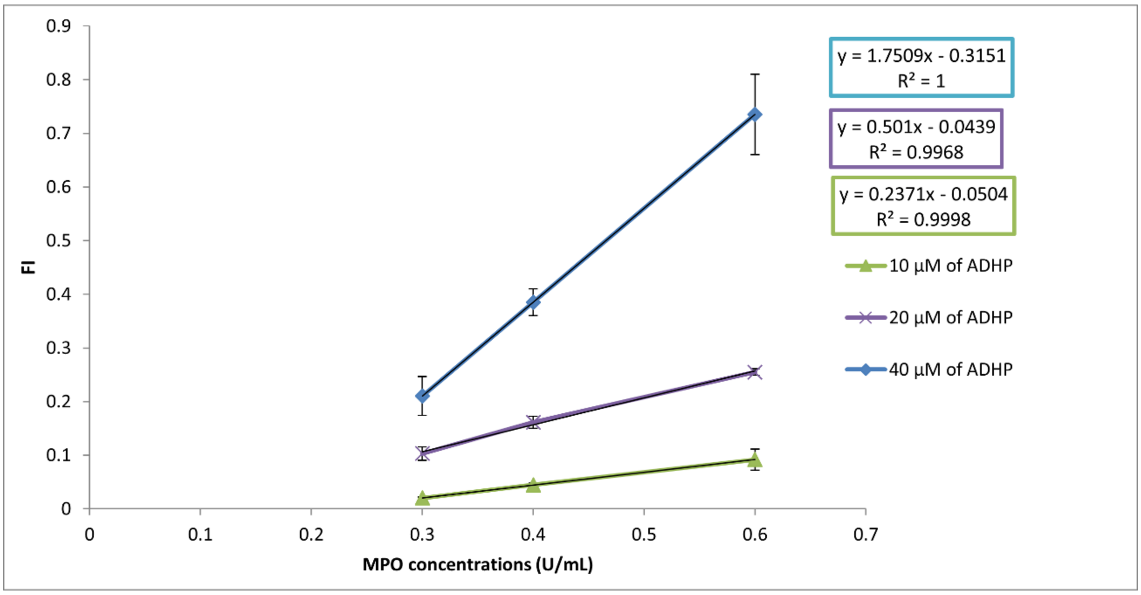

Figure S2. Influence of MPO and ADHP concentration on the analytical signal of the enzymatic reaction.

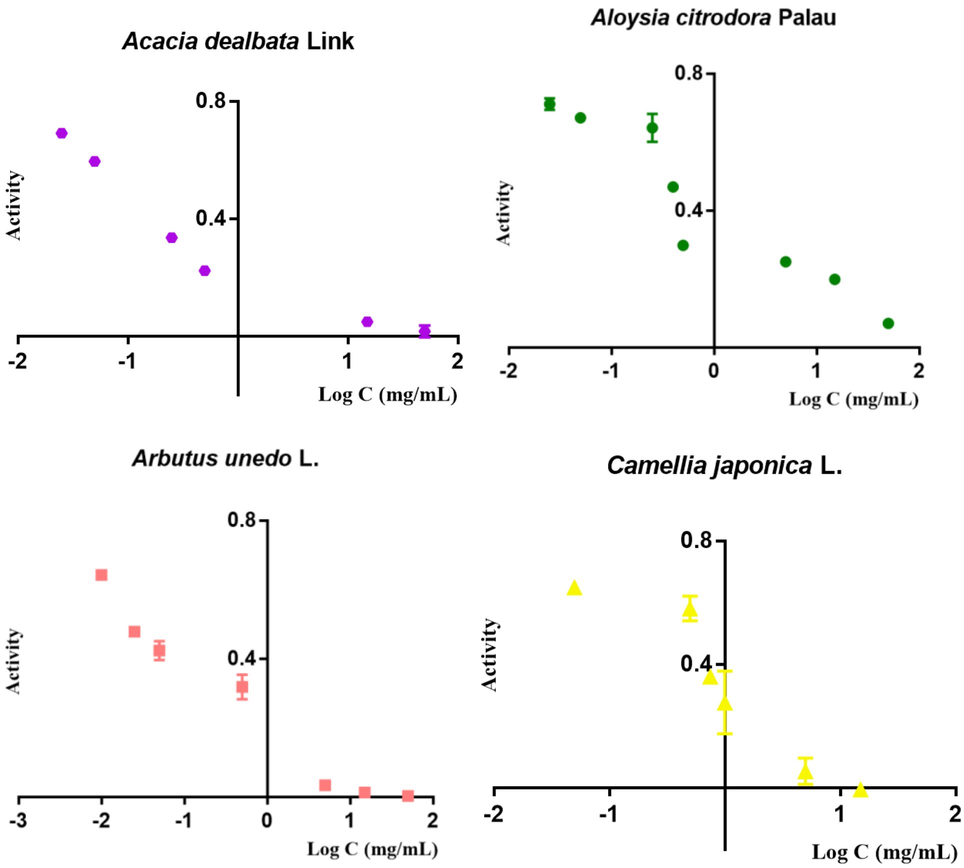

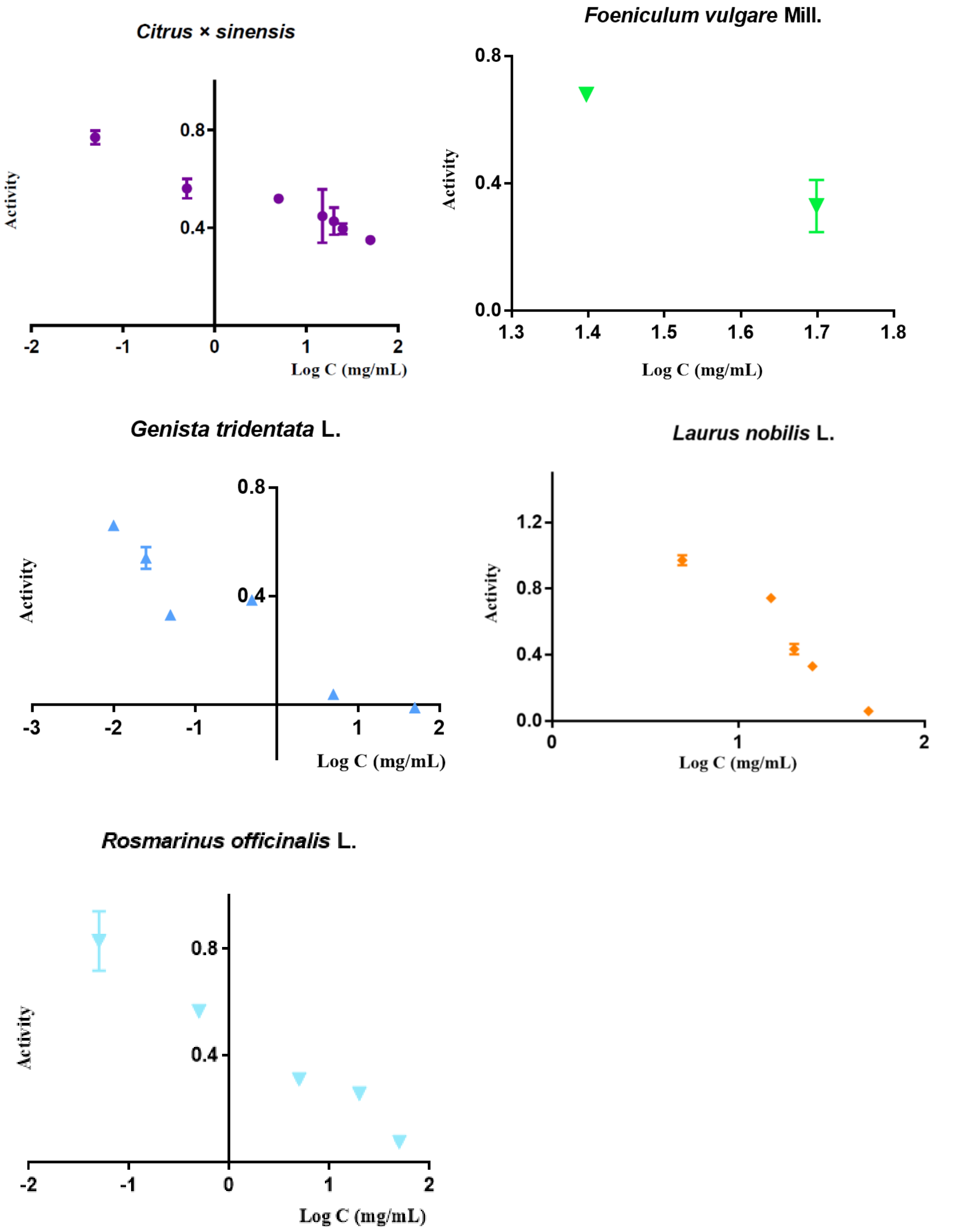

Figure S3. Experimental inhibition data of the plant extracts tested in the MPO inhibition assays in SIA.

Table S2. Analytical cycle developed and optimized in the SIA system.

Step	Position Valve	In/Out	Number of steps	Time (s)	Event
3	2	Out	−320	2.4	Aspiration of ADHP
4	4	Out	−80	1.2	Aspiration of buffer/sample
5	6	Out	−240	1.8	Aspiration of enzyme

6	3	Out	−80	1.2	Aspiration of H_2O_2
7	5	Out	2670	20	Propulsion to the reaction tube
8	5	Out	0	300	5 minutes of stopped flow in the thermostatic bath
9	5	Out	18500	139	Propulsion to the detector
